# Is There an Association between BMI, Height, and Gender and Long-Bone Fractures during Childhood and Adolescence? A Large Cross-Sectional Population Study of 911,206 Subjects

**DOI:** 10.3390/children10060984

**Published:** 2023-05-31

**Authors:** Raphael Lotan, Ran Thein, Barak Gordon, Shay Tenenbaum, Estela Derazne, Dorit Tzur, Arnon Afek, Oded Hershkovich

**Affiliations:** 1 Department of Orthopedic Surgery, Edith Wolfson Medical Center, Affiliated to the Sackler School of Medicine, Holon 5822012, Israel; 2 Department of Orthopedic Surgery, Chaim Sheba Medical Center, Affiliated to the Sackler School of Medicine, Tel Aviv 5262000, Israel; 3 Medical Corps, Israeli Defense Forces, Ramat Gan 91120, Israel; 4 Department of Military Medicine, Faculty of Medicine, Institute for Research in Military Medicine (IRMM), The Hebrew University of Jerusalem, Jerusalem 91120, Israel; 5 Management, Chaim Sheba Medical Center, Affiliated to the Sackler School of Medicine, Tel Aviv 5262000, Israel

**Keywords:** traumatic long-bone fracture (TLF), body mass index (BMI), height, obesity, weight

## Abstract

Introduction: Traumatic long-bone fractures (TLFs) among children and adolescents are relatively common, with morbidity and economic consequences. Obesity has become a significant global concern. Studies have found an association between TLFs and BMI in the past but not in a large cross-sectional population study. Our study objective was to measure the incidence of TLFs in the 17-year-old general population and evaluate its association with BMI, body height, and gender. Methods: Data from a medical database containing all 17-year-old candidates’ records before recruitment into mandatory military service were retrieved as BMI, height, gender, and history of TLFs. Logistic regression models assessed the association between BMI and height to TLFs. Results: The records of 911,206 subjects (515,339 males) were reviewed. In total, 9.65% had a history of TLFs (12.25% and 6.25% for males/females, respectively). Higher BMI was associated with TLF, with a linear trend in the odds ratio (OR) for having TLFs. The strongest association was found between obese females and TLFs (OR = 1.364, *p* < 0.0001). Height was an independent factor positively associated with TLFs. The OR for a TLF in the highest height quintile was 1.238 (*p* < 0.001) for males and 1.411 (*p* < 0.001) for females compared to the lowest quintile. Although TLFs were more common in males, the OR for TLFs was more prominent in females. Conclusions: There is an association between BMI, body height, and TLFs in healthy adolescents. TLFs are more common in males, but the strongest association between overweight and obesity is evident in females.

## 1. Introduction

Instances of childhood fractures are frequently observed in hospital emergency rooms worldwide. According to reports, nearly one out of every four children or adolescents sustains a fracture during childhood. Studies have shown that the incidence of fractures in the pediatric population varies between 12.0 and 36.1/1000 per year. [1,2,3]. These fractures can cause significant morbidity and often require medical treatment, including hospitalization and surgery [1,2]. Fractures of long bones are commonly attributed to low-energy trauma, sports-related injuries, or incidents involving motor vehicles. Large-scale data regarding causes and incidence during childhood and adolescence are sparse [1,3]. Leung et al. reported that 42% of boys and 27% of girls would have a TLF by the time they reached 16 years [1]. Cheng and Shen reported that 6.8% of children were hospitalized due to a fracture [4].

Risk factors associated with long-bone fractures in children and adolescents include age, sex, and physical activity levels. Research indicates that boys risk sustaining long-bone fractures more than girls [2].

Engaging in physical activity is another risk factor for long-bone fractures, as active children tend to participate in activities that may result in bone fractures. Furthermore, research suggests that certain sports, such as basketball, football, soccer, snowboarding, and other contact sports, can increase the likelihood of bone fractures, particularly among male adolescents [5]. Paradoxically, the absence of physical activity or exercise is also considered a risk factor for fractures during childhood [6].

Comorbidities, such as obesity, can heighten the risk of sustaining long-bone fractures [3]. There are several additional risk factors associated with fractures in the pediatric population. These risk factors include poor intrauterine nutrition, very low birth weight, inadequate intake of calcium, vitamin D, and other minerals such as magnesium, zinc, or phosphorus, lactose intolerance, poor nutritional health, obesity, limited exposure to sunlight, lack of family interaction to encourage physical activity and play, and a sedentary lifestyle [5]. Children with genetic diseases, such as children with osteogenesis imperfecta, are at an increased risk of fractures [7]. Children who exhibit low bone mineral density, such as those diagnosed with juvenile idiopathic arthritis, primary or secondary osteomalacia, or other underlying medical conditions, are also more susceptible to sustaining long-bone fractures. These conditions weaken the bones, making them more vulnerable to fracture from low-energy trauma or other injuries [4].

Long-bone fractures can place a significant financial burden on the healthcare system. According to one study, the direct costs associated with pediatric fractures in the United States exceed $2 billion annually [8]. However, indirect costs, such as lost wages and reduced productivity, can also be substantial.

Long-bone fractures among children and adolescents can lead to temporary patient physical and mental morbidity, as children may experience anxiety or depression, which can harm their overall quality of life and well-being. It is, therefore, crucial to provide comprehensive care that addresses not only the physical injury [9] but also the emotional and psychological well-being of the patient [10,11,12].

Preventive measures can be critical in mitigating the incidence of long-bone fractures among children and adolescents. Encouraging weight-bearing physical activities can reduce fracture risk. Adequate nutrition, including a well-balanced diet rich in calcium and vitamin D, can also improve bone health and reduce the risk of fractures. Moreover, practicing appropriate safety measures, such as wearing protective gear during contact sports, can significantly lower the risk of sustaining injuries resulting in long-bone fractures [13]. Additionally, children and adolescents at risk of fractures, such as those with low bone mineral density or osteogenesis imperfecta, can benefit from medical treatment to reduce their risk of fractures.

Obesity has become a significant global concern during childhood and adolescence [8]. The NHNE (National Health and Nutrition Examination) Survey has revealed a significant surge in the prevalence of obesity among children aged 2–19 years in the United States over the past forty years. The findings indicate that the incidence of obesity in this age group has increased nearly fourfold, making it the fastest-growing subpopulation of obese individuals in the country. This alarming trend has garnered widespread concern and prompted calls for increased efforts to combat childhood obesity through public health campaigns, community-based interventions, and policy changes aimed at promoting healthy lifestyles and preventing obesity-related health problems [9,13]. Recent publications have reported similar trends in the Israeli population, indicating an increase in the prevalence of obesity among children and adolescents. However, studies have also revealed that Israeli adolescents tend to exhibit lower obesity rates compared to their American counterparts. Their weight percentiles fall below those reported by the CDC percentile distributions. While this finding may seem encouraging, it is crucial to recognize that even lower levels of obesity can have significant health implications, and efforts to reduce obesity rates among the Israeli pediatric population should continue to be prioritized [14,15].

Manias and colleagues have proposed that a higher BMI may increase the risk of sustaining fractures by creating a mechanical disadvantage during a fall. The added weight and mass associated with a higher BMI can increase the impact force upon landing, potentially leading to more severe injuries. Additionally, body fat distribution may play a role in fracture risk, as excess fat accumulation around the midsection can impact balance and coordination, increasing the likelihood of falls [16,17]. Conversely, while a higher BMI can be associated with increased bone density levels, several mechanisms related to the obese state can also make bones more fragile. For instance, obesity can lead to excess production of inflammatory cytokines, which can trigger bone resorption and increase the risk of fractures. Furthermore, obesity can be associated with comorbidities such as diabetes and metabolic disorders that can impair bone health and increase fracture risk. It is worth noting that while higher bone density levels may seem beneficial, they do not necessarily translate into stronger bones, and other factors must be considered to assess overall bone health [18].

The association between TLFs and body mass index (BMI) has been evaluated in the past [19,20,21,22,23]; it was suggested that low bone density, high BMI, and high adiposity increase fracture risk in males and females. More recent publications showed a higher childhood fracture prevalence in obese children, with reduced treatment outcomes [24,25,26]. Nevertheless, no significant studies explored the association between BMI, body height, gender, and the prevalence of TLFs in a large cross-sectional population study. Other factors associated with fractures during childhood include previous fractures and lower bone mineralization associated with genetic, hormonal, or nutritional factors [16,19]. Therefore, preventive medicine should be the primary goal for at-risk children.

The primary objective of this study is to investigate the prevalence of traumatic long-bone fractures (TLFs) among the general population of 17-year-olds and to evaluate its potential associations with BMI, body height, and gender. Our working hypothesis is that individuals who are overweight may be at a heightened risk of TLFs, and males may be at greater risk than females. By identifying potential risk factors for TLFs among this age group, we hope to inform the development of targeted interventions and preventive strategies that can help mitigate the incidence of these injuries and promote optimal bone health among adolescents.

## 2. Methods

### 2.1. Medical Database

In Israel, all 17-year-old individuals, irrespective of gender, are mandated by law to undergo a comprehensive medical evaluation at a military recruitment center for medical classification before recruitment. Army physicians conduct this evaluation process, including a medical questionnaire filled out by the candidate, a medical report signed by their primary care physician, and a complete anamnesis and physical examination. Candidates may be referred to medical specialists or undergo additional tests if needed. Various parameters, such as body measurements, physical signs, eyesight, and hearing, are carefully evaluated and recorded during the assessment. Each individual is then assigned a unique number on a global medical profile scale and given numerical codes representing their diagnosis and medical status, as defined by the Israeli Defense Forces Regulations for Medical Fitness Determination, which describe different medical conditions. The military physician diagnosed long-bone fractures based on the medical records provided.

For this study, the researchers obtained data from the database of the Israeli Medical Corps, which was authorized for research purposes by the institutional review board of the Israel Defense Forces Medical Corps.

### 2.2. Study Sample

This is a retrospective epidemiological study including 911,206 adolescents evaluated by regional army recruitment centers since 1998 with complete medical data.

### 2.3. Cohort Assignment

In this study, we employed the widely accepted classification system developed by the US Centers for Disease Control and Prevention, which utilizes percentile grading to categorize individuals into different weight categories based on their BMI. Specifically, we classified individuals into one of four categories: underweight (less than the fifth percentile), healthy weight (between the fifth percentile and the 85th percentile), overweight (between the 85th percentile and the 95th percentile), and obese (equal to or greater than the 95th percentile). Furthermore, to better understand the potential associations between body height and the incidence of traumatic long-bone fractures, we stratified the cohort’s height data into five subgroups, each comprising 20% of the total cohort.

All subjects with a documented medical history of long-bone fractures were included in the traumatic long-bone fracture (TLF) group. Fractures involving hands and feet (tarsal\carpal\metacarpal\metatarsal bones, and phalangeal fractures) were excluded. The TLF group was further divided according to the subject’s natural history and pathology: uneventful and complicated fracture healing. The complicated fracture group consisted of subjects with an unexpected healing course, such as fracture re-manipulation, change of treatment, an open fracture, and a documented history of fracture nonunion.

### 2.4. Statistical Analyses

To assess the potential associations between BMI, body height, gender, and the incidence of traumatic long-bone fractures among adolescents in Israel, we employed logistic regression analysis. Specifically, we utilized binary models, where long-bone fractures were considered a dichotomous variable, and multinomial models with no long-bone fracture in the base category. BMI and body height were treated as ordinal variables based on the various subgroups, as mentioned previously, as well as continuous variables.

The results of the logistic regression analyses were reported as odds ratios (OR), 95% confidence intervals (CI), and significance levels. A *p*-value of less than or equal to 0.05 was considered statistically significant. By employing these rigorous statistical methods, we aimed to provide a comprehensive understanding of the potential relationships between BMI, body height, gender, and the incidence of traumatic long-bone fractures among adolescents, which may have important implications for the development of targeted interventions and preventive strategies to mitigate the incidence of these injuries.

## 3. Results

### 3.1. The prevalence of TLFs

The study population included 911,206 young adults (515,339 males and 395,867 females). Cohort characteristics are presented in Table 1. The mean male BMI was 22.03 ± 3.8, and the mean height was 174.1 ± 6.8 cm; these were 21.8 ± 3.7 and 162.1 ± 6.25 cm, respectively, for females. A total of 63,170 (12.25%) males and 24,728 (6.25%) females had a history of long-bone fractures (overall incidence of 9.65%). The overall male incidence of uneventful long-bone fracture healing was 12.21%, 0.043% had complicated fracture healing, and 0.0062% suffered fracture nonunion. The overall female incidence of fracture healing was 6.21%, 0.02%, and 0.002%, respectively. Uneventful TLF was the most common diagnosis, compared to complicated TLF and the rare nonunion group (Table 1). Overall, males suffered 2.55 times more long-bone fractures compared to females. The male gender had an OR of 2.08 (CI = 2.048–2.112, *p* < 0.001) to have a TLF compared to the female gender (Table 2).

### 3.2. Associations between BMI and TLFs

An increased BMI was associated with a history of TLF (Table 2). The ORs for obese and overweight males and females are presented in Table 2: the greater the BMI, the greater the OR for traumatic long-bone fractures among males and females. For males, the ORs for the overweight and obese categories were 1.029 (*p* < 0.001) and 1.044 (*p* < 0.001), respectively. For females, the ORs for the overweight and obese categories were 1.201 (*p* < 0.001) and 1.367 (*p* < 0.001), respectively. Although the incidence of TLF was higher in males, the OR for TLFs was higher in females (OR = 1.364, CI = 1.288–1.445, *p* < 0.0001). Being underweight had a protective effect against TLF in both genders (OR = 0.934, CI = 0.905–0.965, *p* < 0.0001 for males and OR = 0.779, CI = 0.729–0.832, *p* < 0.0001 for females).

### 3.3. Association between Body Height and TLFs

Body height as an independent factor was also positively associated with a history of traumatic long-bone fractures in both genders. The OR for a TLF in the Q5 height quintile compared to Q1 was 1.289 (CI = 1.261–1.319, *p* < 0.001) for both genders combined, 1.238 (CI = 1.205–1.273, *p* < 0.001) for males and 1.411 (CI = 1.356–1.469, *p* < 0.001) for females. There was a linear trend in the OR for both males and females along the height quintiles (Table 2). As with BMI, although the incidence of traumatic long-bone fractures was higher in males, the OR for a TLF was higher for females.

Both BMI and body height showed a linear trend for traumatic long-bone fractures for females and males (Table 3).

## 4. Discussion

This study found that the younger than 17 years’ cross-sectional population incidence of traumatic long-bone fracture was 12.21% for males and 6.21% for females. The cohort’s overall incidence of long-bone fractures was 9.65%. We found an association between BMI and height and traumatic long-bone fractures with a linear trend across weight categories and height quintiles.

Fractures in childhood are widespread and represent one of the most common injuries present in hospital emergency rooms globally. Indeed, statistics indicate that as many as one in four children and adolescents may experience a fracture at some point during their childhood years. The incidence rates of fractures in the pediatric age group have varied between 12.0 and 36.1 per 1000 children per year, highlighting the significant public health burden that these injuries represent [1,2,3]. Long-bone fractures are frequently caused by motor vehicle accidents, sports injuries, or low-energy trauma, such as falls. However, despite their high incidence rates, there is a notable lack of large-scale data on the specific causes and incidence of these fractures during childhood and adolescence [1,3,4].

Risk factors for long-bone fractures in children and adolescents include age, sex, and physical activity. Boys are at a higher risk of long-bone fractures than girls [2]. Engaging in physical activity is a well-known risk factor for long-bone fractures, as active children are more likely to participate in activities that can result in falls or other types of trauma. Certain sports, including basketball, football, soccer, snowboarding, and contact sports such as football and rugby, have been linked to a higher incidence of fractures in the pediatric age group. Interestingly, however, the absence of any physical activity or exercise is also recognized as a risk factor for fractures in childhood, likely due to decreased bone density and strength resulting from a sedentary lifestyle. In addition to physical activity, specific comorbidities, such as obesity, have also increased the risk of long-bone fractures in children and adolescents [3].

There are several established risk factors for fractures in the pediatric population, including poor intrauterine nutrition, very low birth weight, low intake of essential nutrients such as calcium, vitamin D, magnesium, zinc, or phosphorus, lactose intolerance, and poor nutritional health. Additionally, obesity has been identified as a risk factor for fractures, likely due to increased stress on bones and decreased bone density. Low exposure to sunlight, which is necessary for synthesizing vitamin D, has also been associated with a higher incidence of fractures in children and adolescents.

The financial burden of long-bone fractures on the healthcare system can be substantial. Hospitalization, surgery, and follow-up care can be expensive, depending on fracture severity and the hospitalization required. One study estimated that the direct cost of pediatric fractures in the United States was over USD 2 billion annually [6]. These costs include hospitalization, surgery, and follow-up care, with indirect costs that can also be substantial, such as lost wages and reduced productivity.

Obesity has become a significant global concern during childhood and adolescence [8]. Recent research based on the NHNE (National Health and Nutrition Examination) Survey reported a concerning trend regarding obesity rates in children and adolescents in the United States. The survey found that over the past forty years, the prevalence of obesity among individuals aged 2–19 years has increased significantly, nearly fourfold. This trend is alarming due to this being the fastest-growing subpopulation in the United States [9,13]. Recent publications have reported similar patterns in the Israeli population, although Israeli adolescents appear to have lower obesity rates, with lower percentiles compared to the CDC percentile distribution [14,15].

Manias et al. suggested that a higher BMI would result in mechanical disadvantage during a fall, causing a higher fracture rate [16] and lower levels of weight-bearing physical activity, negatively affecting coordination or balance, which can increase the risk of falling [19]. The association between TLFs and body mass index (BMI) has been evaluated in the past [19,20,21,22,23]; it was suggested that low bone density, high BMI, and high adiposity increase fracture risk in males and females. More recent publications showed a higher childhood fracture prevalence in obese children with reduced treatment outcomes [24,25,26]. Nevertheless, no significant studies explored the association between BMI, body height, gender, and the prevalence of TLFs in a large cross-sectional population study.

The findings of this study indicate that among individuals younger than 17 years old, the incidence of traumatic long-bone fracture is 12.21% for males and 6.21% for females in the cross-sectional population. The overall incidence of long-bone fractures within the cohort was 9.65%. Moreover, a significant association was observed between BMI and height, showing a linear trend across different weight categories and height quintiles in relation to traumatic long-bone fractures. The association between BMI and fractures has been investigated in the past, but studies were based on relatively small cohorts and there is a lack of diversity in the populations studied; a significant part of this data came from a single research group from New Zealand [19,21,27,28].

An association between body height and fractures in the general population has not been reported. There are a few reports regarding the association between elderly femoral neck fractures and other osteoporotic fractures to body height [29] but not in young and healthy populations. Our findings revealed a correlation between taller height and TLFs, with a linear trend amongst height quantiles. Probable explanations can include reduced agility, coordination, or physical activity attendance in tall individuals. Another possible explanation is the different mechanical forces acting on long bones during the fall, including moment arm variation by length [30].

Overweight and obesity are increasing in Israel [31] and worldwide [32]. Being overweight or obese is becoming a significant risk factor for various medical conditions [33]. Our findings support the call for population education and fundamental lifestyle modifications to reduce the prevalence of obesity already in childhood age. The same interventions should also aid in traumatic long-bone fracture reduction during childhood and adolescence and its financial burden on the health care systems.

This study’s limitations include its epidemiological nature, excluding causality. The fracture mechanism and date were unavailable, as was information regarding other coexisting skeletal or soft tissue injuries. Fractures were divided into three broad categories (uneventful TLFs, complicated TLFs, and nonunions) according to the military medical board classification (RMFD) and not by an orthopedic classification, such as the AO classification (Arbeitsgemeinschaft für Osteosynthesefragen classification). Clustering upper and lower long-bone fractures into a single group generalizes potentially different etiologies and mechanisms of injury. Another limitation of this large cross-sectional population study was the lack of data regarding the age at which the traumatic long-bone fracture occurred, as most girls reach an adult bone mass by the age of 14–16, while males reach an adult bone mass after age 16, so a certain proportion of the fractures may have occurred in subjects that could be considered adults. Finally, we do not have information on cohort subjects’ physical activities, which can influence the individual inherent risk of TLF.

Additional investigations of the biomechanical and physiological aspects are necessary to provide insights into the underlying mechanisms of the association between body measurements and traumatic long-bone fractures to enhance our comprehension of the effects of weight reduction on alleviating the fracture burden in the pediatric age group.

## 5. Conclusions

This study represents a significant contribution to the field as one of the largest cross-sectional population studies. The findings confirm the previously suggested but not well-established associations between BMI, body height, and traumatic long-bone fractures.

## Figures and Tables

**Table 1:** This study’s cohort characteristics.

Males			
	No TLFs (%)	Uneventful TLFs (%)	Complicated TLFs (%)
BMI			
Underweight	8.1	7.5	8.5
Healthy weight	87.8	75.1	73.3
Overweight	9.8	10.2	9.5
Obese	6.7	7.2	8.7
Height quintiles (Q)			
Q1 (130–168 cm)	20.9	18.7	20.5
Q2 (169–172 cm)	20.9	20.3	20.5
Q3 (173–176 cm)	23.2	23.4	26.4
Q4 (177–180 cm)	18.9	19.7	15
Q5 (181–210 cm)	16.2	18	17.6
**Females**			
	**No TLFs (%)**	**Uneventful TLFs (%)**	**Complicated TLFs (%)**
BMI			
Underweight	5	3.9	4.8
Healthy weight	80.5	78.6	77
Overweight	10.4	12	10.5
Obese	4.1	5.5	7.7
Height quintiles (Q)			
Q1 (130–168 cm)	23.6	19.7	24
Q2 (169–172 cm)	18.1	17.5	10.5
Q3 (173–176 cm)	24.3	24.3	19.3
Q4 (177–180 cm)	15.5	16.8	22.2
Q5 (181–210 cm)	18.5	21.7	24

**Table 2:** Odds ratio and 95% confidence interval for TLF by severity with BMI and height categories ^a^.

(a) Both genders combined.						
	Binary Logistic Regression			Multinomial Logistic Regression		
	All TLFs			Uneventful TLFs		
	OR	95% CI	*p*-Value	OR	95% CI	*p*-Value
BMI ^b^						
Underweight	0.906	0.880–0.932	<0.001	0.905	0.880–0.931	<0.001
Healthy weight	1				1	
Overweight	1.080	1.056–1.105	<0.001	1.080	1.056–1.105	<0.001
Obese	1.111	1.080–1.143	<0.001	1.109	1.078–1.141	<0.001
Height quintiles ^b^ (Q)						
Q1 (130–168 cm)	1			1		
Q2 (169–172 cm)	1.102	1.078–1.127	<0.001	1.103	1.079–1.128	<0.001
Q3 (173–176 cm)	1.142	1.118–1.167	<0.001	1.143	1.118–1.168	<0.001
Q4 (177–180 cm)	1.198	1.171–1.226	<0.001	1.199	1.172–1.227	<0.001
Q5 (181–210 cm)	1.289	1.260–1.318	<0.001	1.289	1.261–1.319	<0.001
Gender	2.079	2.048–2.112	<0.001	2.080	2.048–2.112	<0.001
**(b) Males**						
	**Binary Logistic Regression**			**Multinomial Logistic Regression**		
	**All TLFs**			**Uneventful TLFs**		
	**OR**	**95% CI**	***p*-Value**	**OR**	**95% CI**	***p*-Value**
BMI ^b^						
Underweight	0.935	0.906–0.906	<0.001	0.934	0.905–0.965	<0.001
Healthy weight	1			1		
Overweight	1.029	1.001–1.058	0.045	1.029	1.001–1.058	0.044
Obese	1.044	1.011–1.079	0.010	1.043	1.009–1.078	0.012
Height quintiles ^b^ (Q)						
Q1 (130–168 cm)	1			1		
Q2 (169–172 cm)	1.078	1.050–1.107	<0.001	1.078	1.050–1.107	<0.001
Q3 (173–176 cm)	1.117	1.088–1.146	<0.001	1.117	1.088–1.146	<0.001
Q4 (177–180 cm)	1.158	1.127–1.189	<0.001	1.159	1.129–1.191	<0.001
Q5 (181–210 cm)	1.238	1.204–1.272	<0.001	1.238	1.205–1.273	<0.001
**(c) Females**						
	**Binary Logistic Regression**			**Multinomial Logistic Regression**		
	**All TLFs**			**Uneventful TLFs**		
	**OR**	**95% CI**	***p*-Value**	**OR**	**95% CI**	***p*-Value**
BMI ^b^						
Underweight	0.780	0.730–0.833	<0.001	0.779	0.729–0.832	<0.001
Healthy weight	1			1		
Overweight	1.201	1.10.54–1.250	<0.001	1.201	1.154–1.250	<0.001
Obese	1.367	1.290–1.447	<0.001	1.364	1.288–1.445	<0.001
Height quintiles ^b^ (Q)						
Q1 (130–168 cm)	1					
Q2 (169–172 cm)	1.159	1.111–1.209	<0.001	1.162	1.114–1.212	<0.001
Q3 (173–176 cm)	1.202	1.156–1.250	<0.001	1.205	1.159–1.252	<0.001
Q4 (177–180 cm)	1.305	1.251–1.362	<0.001	1.305	1.250–1.362	<0.001
Q5 (181–210 cm)	1.410	1.355–1.468	<0.001	1.411	1.356–1.469	<0.001

^a^ OR, odds ratio; CI, confidence interval. All OR and CI values are in comparison to normal weight and to height Q1. Results are given as OR (95% CI). ^b^ calculated as a continuous variable.

**Table 3:** Linear trends for uneventful TLFs concerning BMI and body height.

	Uneventful TLFs			All TLFs		
	b	*p*	Adjusted R^2^	b	*p*	Adjusted R^2^
**Male**						
BMI	0.036	0.048	0.861	0.035	0.05	0.854
Height	0.056	0.001	0.976	0.056	0.001	0.976
**Female**						
BMI	0.196	0.002	0.994	0.196	0.002	0.993
Height	0.097	0.002	0.965	0.097	0.002	0.965

## Data Availability

The complete data are available under a confidentiality restriction.

## Abbreviations

Traumatic long-bone fracture (TLF), body mass index (BMI).
